# Nuclear Signaling of Plant MAPKs

**DOI:** 10.3389/fpls.2018.00469

**Published:** 2018-04-11

**Authors:** Jean Bigeard, Heribert Hirt

**Affiliations:** ^1^Institute of Plant Sciences Paris-Saclay IPS2, Centre National de la Recherche Scientifique, Institut National de la Recherche Agronomique, Université Paris-Sud, Université Evry, Université Paris-Saclay, Orsay, France; ^2^Institute of Plant Sciences Paris-Saclay IPS2, Paris Diderot, Sorbonne Paris-Cité, Orsay, France; ^3^Center for Desert Agriculture, King Abdullah University of Science and Technology, Thuwal, Saudi Arabia

**Keywords:** nucleus, mitogen-activated protein kinase, phosphorylation, signaling, biotic stress, abiotic stress, development

## Abstract

Mitogen-activated protein kinases (MAPKs) are conserved protein kinases in eukaryotes that establish signaling modules where MAPK kinase kinases (MAPKKKs) activate MAPK kinases (MAPKKs) which in turn activate MAPKs. In plants, they are involved in the signaling of multiple environmental stresses and developmental programs. MAPKs phosphorylate their substrates and this post-translational modification (PTM) contributes to the regulation of proteins. PTMs may indeed modify the activity, subcellular localization, stability or trans-interactions of modified proteins. Plant MAPKs usually localize to the cytosol and/or nucleus, and in some instances they may also translocate from the cytosol to the nucleus. Upon the detection of environmental changes at the cell surface, MAPKs participate in the signal transduction to the nucleus, allowing an adequate transcriptional reprogramming. The identification of plant MAPK substrates largely contributed to a better understanding of the underlying signaling mechanisms. In this review, we highlight the nuclear signaling of plant MAPKs. We discuss the activation, regulation and activity of plant MAPKs, as well as their nuclear re-localization. We also describe and discuss known nuclear substrates of plant MAPKs in the context of biotic stress, abiotic stress and development and consider future research directions in the field of plant MAPKs.

## Introduction

In nature, plants constantly face changing conditions of both biotic and abiotic origin. To resist or tolerate these stresses, plants notably reprogram adequately the expression of their genes. This transcriptional reprogramming occurs downstream of a complex network of signaling pathways that is activated after signal perception at the cell surface. Understanding the signaling mechanisms allowing the transduction of the signal from the plasma membrane to the nucleus is an enormous challenge. This is especially true knowing the nucleus structure which includes a double membrane system that is punctuated by nuclear pores that allow the active transport or diffusion of molecules between the cytosolic and the nuclear compartments (Meier, 2007; Tamura and Hara-Nishimura, 2013). Diffusion into the nucleus is usually ineffective for molecules larger than 20–40 kDa (Fried and Kutay, 2003).

Post-translational modifications (PTMs) constitute an important mechanism of protein regulation. Most PTMs are catalyzed by specific enzymes and are often reversible. They may activate/inhibit the activity of an enzyme, change the subcellular localization or stability of a protein and allow or prevent trans-interactions (Lothrop et al., 2013; Bigeard et al., 2014). Several hundred different PTMs have been reported and protein phosphorylation is probably the most abundant PTM found in eukaryotes (Khoury et al., 2011; Minguez et al., 2012; Olsen and Mann, 2013). Protein phosphorylation, mediated by protein kinases and removed by protein phosphatases, occurs predominantly on serine, threonine, and tyrosine residues. Plant genomes code for numerous protein kinases compared to other eukaryotes. For instance, about 1,000 and 1,400 genes are predicted to code for protein kinases in *Arabidopsis thaliana* (Arabidopsis) and *Oryza sativa* (rice), respectively, while the genomes of *Homo sapiens* and *Saccharomyces cerevisiae* code for about 500 and 120 protein kinases, respectively (The Arabidopsis-Genome-Initiative, 2000; Lander et al., 2001; Venter et al., 2001; Wang et al., 2003; Dardick et al., 2007). Among them, the mitogen-activated protein kinases (MAPKs) are conserved protein kinases among eukaryotes (Doczi et al., 2012; Lehti-Shiu and Shiu, 2012) and constitute signaling modules where MAPK kinase kinases (MAPKKKs) activate MAPK kinases (MAPKKs) which in turn activate MAPKs. In Arabidopsis, there are 20 MAPKs, 10 MAPKKs, and 60 MAPKKKs (MAPK-Group, 2002), and they are implicated in the signaling of multiple environmental stresses and developmental programs (Colcombet and Hirt, 2008; Rodriguez et al., 2010).

The identification of plant MAPK substrates is necessary to fully understand MAPK function and decipher the signaling mechanisms leading notably to the transcriptional reprogramming. Several hundred putative MAPK substrates have been identified *via* targeted experiments and more systematic approaches, such as phosphoproteomics and protein array screening (Feilner et al., 2005; Popescu et al., 2009; Hoehenwarter et al., 2013; Rayapuram et al., 2017). Among them, some were more extensively validated as MAPK targets, qualifying them as *bona fide* MAPK substrates. Actually, a large majority of the currently known *bona fide* plant MAPK substrates are transcription factors, indicating the preponderant involvement of plant MAPKs in nuclear signaling.

In this review, we first discuss the activation, regulation and activity of plant MAPKs, and their nuclear re-localization. We then present and discuss known nuclear substrates of plant MAPKs involved in biotic stress, abiotic stress and development, thereby putting forward the nuclear signaling of plant MAPKs. We finally discuss several highlights and unresolved questions regarding MAPK substrates and functions.

## MAPK activation and regulation

Based on phylogenetic methods, plant MAPKs are divided into four groups (A to D) (MAPK-Group, 2002) and are characterized by the conserved TxY consensus motif in their activation loop (T-loop) which, when doubly phosphorylated by MAPKKs on the threonine and tyrosine residues, activate MAPKs (Anderson et al., 1990; Payne et al., 1991; MAPK-Group, 2002). Sequence comparison of this motif allowed classifying MAPKs into two subtypes: the TEY subtype gathers groups A, B, and C; the TDY subtype forms the more distant group D. Most MAPKs (groups A, B, and C, but not group D) also have a CD domain in their C-terminal region, which is a docking site for MAPKKs, MAPK phosphatases, and substrates, and corresponds to the amino acid sequence [LH][LHY]Dxx[DE]xx[DE]EPxC (Tanoue et al., 2000). On the other side, most MAPKKs, MAPK phosphatases and substrates have a putative MAPK docking site (D-site) which corresponds to the amino acid sequence [K/R][K/R][K/R]x(1–5)[L/I]x[L/I] and which binds the MAPK CD domain (Bardwell and Thorner, 1996; MAPK-Group, 2002; Bardwell, 2006).

Although the mechanism of plant MAPK activation is consensual, the kinetics of MAPK activation is variable and depends both on the upstream signaling events leading to MAPK activation and on the mechanisms of deactivation or turn-over of the MAPKs. For instance, the Arabidopsis MPK3, MPK4, MPK6, and MPK11 are activated by microbe-associated molecular patterns (MAMPs), such as the 22 amino acid long epitope flg22 from the N terminus of *Pseudomonas aeruginosa* flagellin (Gómez-Gómez and Boller, 2000), within 1 to 2 min leading to a peak of activity around 10–15 min which then rapidly decreases (Nuhse et al., 2000; Zipfel et al., 2006; Denoux et al., 2008; Ranf et al., 2011; Bethke et al., 2012; Frei Dit Frey et al., 2014). Besides, in the absence of MAMP-triggered immunity (MTI) but upon expression induction of a single effector, such as AvrRpt2 effector from *Pseudomonas syringae*, Arabidopsis MPK3 and MPK6 activation is detected at 3 h after induction and lasts for at least 7 h (Tsuda et al., 2013). Similar results are obtained when Arabidopsis leaves are infiltrated with *P. syringae* expressing AvrRpt2 (Underwood et al., 2007; Tsuda et al., 2013). As another example, the Arabidopsis MPK1 and MPK2 are activated by wounding with a peak around 2 h (Ortiz-Masia et al., 2007). As H_2_O_2_, jasmonic acid (JA) and abscisic acid (ABA) are involved in the wounding response, the authors also examined the effect of these molecules on MPK1 and MPK2 activity. The two MAPKs are activated by 5 mM H_2_O_2_ already after 15 min (first time point tested). Their results also show that 50 μM JA induces the activation of the MAPKs already after 30 min (first time point tested) and their activity peaks in both cases at 1 h after treatment. Finally, 100 μM ABA activates MPK1 and MPK2 already after 15 min (first time point tested), with a peak at 2 h, and an activity which returns to basal levels by 8 h. Actually, Arabidopsis MPK7, which belongs to the group C MAPKs like MPK1 and MPK2, is also activated by ABA (50 μM) with different time points tested showing a peak at 4 h (Danquah et al., 2015).

MAPK deactivation occurs *via* dephosphorylation of their activation loop by protein phosphatases (Lee and Ellis, 2007; Brock et al., 2010; Lumbreras et al., 2010; Umbrasaite et al., 2010; Galletti et al., 2011; Carrasco et al., 2014). These phosphatases belong to different families: protein tyrosine phosphatases (PTPs), serine/threonine phosphatases (PSTPs), and dual-specificity (Ser/Thr and Tyr) phosphatases (DSPs) (Andreasson and Ellis, 2010; Bartels et al., 2010). They are crucial because they regulate the intensity and duration of MAPK activation and thus their signaling output. For instance, we mentioned above the difference of activation of Arabidopsis MPK3 and MPK6 by MAMPs (transient activation with peak around 15 min) and by pathogen effectors (activation for several hours). This difference of MAPK activation could be due to MAPK phosphatases which are active in MTI, rapidly dephosphorylating MAPKs, but inactive in effector-triggered immunity (ETI), resulting in the sustained activation of MAPKs (Tsuda et al., 2013). Interestingly, the abundance and activity of these phosphatases are often regulated by the MAPKs they dephosphorylate, thus forming a feed-back mechanism (Bartels et al., 2010; Park et al., 2011; González Besteiro and Ulm, 2013).

MAPK protein turn-over should also contribute to their kinetics of activation. However, this mechanism is scarcely documented, certainly because of the rapidity and efficiency of the phosphatase-dependent mechanism. For example, it was reported that Arabidopsis MPK3 and MPK6 abundance is down-regulated at the protein level by the RAF-like MAPKKK EDR1 (Zhao et al., 2014).

## MAPK activity and mechanisms of specificity

MAPKs are proline-directed serine/threonine kinases, meaning that the presence of a proline at position +1 of the phosphorylated site is required for substrate phosphorylation. Preferential positions of other amino acids were also observed, such as a proline at position −2 and a basic amino acid at position +2 (Berriri et al., 2012; Sorensson et al., 2012). The S/T-P motif thus constitutes the minimal MAPK phosphorylation site motif, and indeed all MAPK protein substrates identified to date confirmed the requirement of this minimal motif. However, the S/T-P motif is present in about 80% of all proteins, indicating that other mechanisms are also necessary to select a given protein as a MAPK substrate (Bardwell, 2006).

The presence of MAPK docking motifs in proteins contributes to the specificity of MAPK substrates and most MAPK substrates indeed have a D-site for interaction with the MAPK CD domain. Generally, D-sites in MAPK substrates are located <100 amino acids upstream (N-terminal) from the MAPK phosphorylation site (Biondi and Nebreda, 2003; Ubersax and Ferrell, 2007). Besides D-sites, other docking motifs were also identified such as the amino acid sequence FxFP (DEF motif) and its variants, and the amino acid sequence LxxRR (Jacobs et al., 1999; Biondi and Nebreda, 2003; Sheridan et al., 2008). The combination of docking motifs may stabilize MAPK-substrate interactions and increase MAPK specificity and efficiency of phosphorylation.

In addition to docking motifs, MAPK specificity can also be brought about *via* adaptors or scaffolds which are proteins acting as organizing platforms that assemble the protein kinase and the substrate in the same complex (Zeke et al., 2009; Good et al., 2011). This mechanism is widely used in mammals and yeasts where notably scaffold proteins recruit the three kinases of MAPK modules (McDonald et al., 2000; Good et al., 2009; Zeke et al., 2009). Surprisingly, very few scaffold proteins have been identified to date in the context of plant MAPK signaling. The first clear plant MAPK scaffold is Arabidopsis RACK1 (Cheng et al., 2015). *P. aeruginosa*-secreted proteases activate a pathway involving heterotrimeric G-protein complexes and the MAPK module MEKK1-MKK4/MKK5-MPK3/MPK6. In this signaling pathway, RACK1 functions as a scaffold, binding to the Gβ subunit of the heterotrimeric G-protein complex as well as to all three tiers of the MAPK module. Actually, MEKK1 was shown quite early to be able to interact with MKK1 and MKK2 but also MPK4 by yeast two-hybrid assays, suggesting that MEKK1 may be a scaffold protein (Ichimura et al., 1998). In addition, it was later shown that a kinase-impaired version of MEKK1, mutated in the ATP binding site (K361M), was able to rescue the *mekk1* mutant dwarf phenotype, suggesting that MEKK1 would play a structural role that is independent of its protein kinase activity (Suarez-Rodriguez et al., 2007). This observation could reinforce a scaffold function for MEKK1. However, it is also possible that MEKK1^K361M^ possesses a residual kinase activity that is sufficient to perform its normal functions, and there is still no evidence that MEKK1 and MPK4 can interact *in planta*. In tomato, the 14-3-3 protein TFT7 was also suggested to function as a scaffold because TFT7 can form a homodimer and it can interact with both the MAPKKK SlMAPKKK alpha and the MAPKK SlMKK2 (Oh and Martin, 2011). Similarly, the Arabidopsis transcription factor MYB44 is able to homodimerize and can interact with the nuclear sub-pools of both MKK4 and MPK3, suggesting that MYB44 could be a MAPK scaffold (Persak and Pitzschke, 2013).

Finally, co-expression and co-localization of MAPKs and their substrates in the same structures or subcellular compartments constitutes another way to regulate specificity (Kholodenko, 2006; Ubersax and Ferrell, 2007; Menges et al., 2008). The co-localization mechanism was shown notably for mammalian extracellular signal-regulated kinase (ERK) MAPKs (Robinson et al., 1998; Whitehurst et al., 2004). Dynamic nucleocytoplasmic shuttling of plant MAPKs is also observed (see the corresponding section below), probably contributing to specificity. For instance, the nucleocytoplasmic shuttling of mammalian ERK1 is slower than ERK2 and this mechanism significantly contributes to the differential capacity of ERK1 and ERK2 to produce signaling output (Marchi et al., 2008).

Substrate phosphorylation by protein kinases may have direct molecular effects and functional protein consequences. Phosphorylation may modify the global structure of a protein by conformational changes and may also alter interactions with other molecules (protein, DNA, RNA, etc.). For instance, the dual phosphorylation of the TxY motif changes the protein conformation of MAPKs rendering them active (Canagarajah et al., 1997), and the negative charge brought about by one or multiple phosphorylation events constitutes an interaction regulation mechanism (Cohen, 2000; Holmberg et al., 2002; Serber and Ferrell, 2007). These molecular changes may have multiple functional consequences. Phosphorylation may indeed activate or inhibit an enzyme activity, allow or prevent interaction between molecules, modify subcellular localization, or modify protein stability. To assess the functional roles of a phosphorylation event, the usual strategy consists in mutating the phosphorylated amino acid to a non-phosphorylatable amino acid (e.g., substitution of Ser to Ala) and/or to a phospho-mimicking amino acid (e.g., substitution of Ser to Asp), and in expressing this mutated substrate in a knock-out line for the corresponding gene, ideally under the control of its own promoter.

## From putative to genuine MAPK substrates

Usually, putative MAPK substrates are initially identified from systematic approaches, such as phosphoproteomics, protein array or yeast two-hybrid screening. However, much more evidence is necessary to qualify a protein as a genuine MAPK substrate.

At the biochemical level, the protein should be phosphorylated *in vitro* by the MAPK. This may be achieved for instance by a classical kinase assay using radiolabeled ATP or by a non-radioactive kinase assay followed by mass spectrometry analysis. Whatever the approach, the identification of the precise phosphorylation site(s) on the protein is required. A proof of the existence *in planta* of the phosphosite(s) identified *in vitro* is necessary as well. This proof may be found in databases such as PhosPhAt (Durek et al., 2010) that gather phosphoproteomic results, or a targeted experiment may be required to identify the phosphosite(s) *in planta*. An interaction *in vivo* between the protein and the MAPK should also be demonstrated. This may be achieved for example *via* bimolecular fluorescence complementation or by co-immunoprecipitation assays. Complementary approaches, showing the interaction between the protein kinase and its substrate can also be instructive, for instance by yeast two-hybrid or pull-down assays.

Genetically, it is expected that the protein phosphorylation disappears, or at least decreases in case of MAPK functional redundancy, in a *mapk* knock out line, and increases in a constitutively active or overexpressing *MAPK* mutant. We mentioned above the phospho-mutant versions of the candidate substrate (e.g., Ser to Ala and Ser to Asp phosphorylation site mutants). It may also be possible that these phosphosite mutant versions recapitulate the phenotype of the *mapk* knock out and/or the constitutively active or overexpressing *MAPK* mutant.

Overall, both biochemical and genetic evidence is thus required to designate a protein as a *bona fide* MAPK substrate, and obviously, several independent lines of evidence enhance the confidence for such an assumption.

## Nuclear localization of MAPKs

Only a small number of strictly nuclear protein kinases are known in plants in comparison to animals (Dahan et al., 2010). The majority of known nuclear plant protein kinases are indeed also localized in the cytosol. This is the case for the majority of plant MAPKs. The spatial organization of MAPKs, and more generally MAPK modules, and the signaling complexity was discussed elsewhere (Šamajová et al., 2012). Notably, the nuclear localization of MAPKs may be explained by the presence of nuclear sorting signals, i.e., nuclear localization signals (NLSs) or nuclear export signals (NESs), on the MAPKs themselves and/or on their substrates and regulatory proteins (e.g., activating MAPKKs and inactivating MAPK phosphatases) (Šamajová et al., 2012).

The first subcellular localization of plant MAPKs was determined in onion and pepper with the homologs of the tobacco NTF6 MAPK which displayed both a cytosolic and nuclear localization (Préstamo et al., 1999). Since then, the subcellular localization of a large number of MAPKs from different plant species was determined. In Arabidopsis, AtMPK3 is located in the cytosol, in the nucleus, and also associated to membranes (Ahlfors et al., 2004; Brock et al., 2010; Umbrasaite et al., 2010; Maldonado-Bonilla et al., 2013; Persak and Pitzschke, 2013; Pitzschke et al., 2014). AtMPK4 localizes to the cytosol, the nucleus, the plasma membrane, the cell plate and to microtubules (Schweighofer et al., 2007; Gao et al., 2008; Brock et al., 2010; Kosetsu et al., 2010; Umbrasaite et al., 2010; Beck et al., 2011). AtMPK6 was found in the cytosol, the nucleus, the plasma membrane, on mitotic microtubules and on secretory trans-Golgi network vesicles (Ahlfors et al., 2004; Schweighofer et al., 2007; Yoo et al., 2008; Brock et al., 2010; Muller et al., 2010; Umbrasaite et al., 2010; Maldonado-Bonilla et al., 2013; Persak and Pitzschke, 2013; Sethi et al., 2014). AtMPK3, AtMPK4, and AtMPK6 have been the most extensively studied Arabidopsis MAPKs, but some data also exist for other Arabidopsis MAPKs. For instance, AtMPK11 is located in both the nucleus and cytosol (Carrasco et al., 2014), and AtMPK12 is localized in both the nucleus and cytosol of guard cells (Jammes et al., 2009).

For the following plant species, we indicate in brackets the probable Arabidopsis orthologs of the cited MAPKs. In tobacco, the MAPKs NtWIPK (AtMPK3 ortholog), NtSIPK (AtMPK6 ortholog), NaMPK4 (AtMPK4/AtMPK11 ortholog), NTF4, and NTF6 all localize to both the nucleus and cytosol (Menke et al., 2005; Yap et al., 2005; Ishihama et al., 2011; Hettenhausen et al., 2012). In rice, OsMPK6 (AtMPK6 ortholog) is located only in the nucleus (Shen et al., 2010). Likewise, OsBWMK1 (AtMPK8/AtMPK9 ortholog) seems to localize exclusively to the nucleus (Cheong et al., 2003), however Koo et al. later reported that alternative splicing of *OsBWMK1* generates three variants with different subcellular localizations: OsBWMK1Long and OsBWMK1Middle localize predominantly to the cytosol, while OsBWMK1Small primarily localizes in the nucleus (Koo et al., 2007). In cotton, GhMPK2 (AtMPK2 ortholog), GhMPK7 (AtMPK7/AtMPK14 ortholog), GhMPK11 (AtMPK4/AtMPK11 ortholog), and GhMPK16 (AtMPK16 ortholog) localize predominantly in the nucleus (Shi et al., 2010, 2011; Zhang et al., 2011; Wang et al., 2016), while GhMPK6a (AtMPK6 ortholog) and GhMPK20 (AtMPK20 ortholog) are present in both the cytosol and nucleus (Li et al., 2013; Wang et al., 2017). In canola, BnaMPK3, BnaMPK5, BnaMPK6, and BnaMPK9 (AtMPK3, AtMPK5, AtMPK6, and AtMPK9 ortholog, respectively) all localize in both the cytosol and nucleus (Liang et al., 2013). In maize, ZmMPK7 (AtMPK7/AtMPK14 ortholog) and ZmMPK17 (AtMPK15 ortholog) are located in the nucleus (Zong et al., 2009; Pan et al., 2012), while ZmMPK3 (AtMPK3 ortholog) is present in both the cytosol and nucleus (Wang et al., 2010). In wheat, TMPK3/WCK-1 (AtMPK3 ortholog) and TMPK6 (AtMPK6 ortholog) are present predominantly in the nucleus (Zaïdi et al., 2010). In Medicago species, alfalfa SIMK (AtMPK6 ortholog) locates predominantly in the nucleus (Munnik et al., 1999; Samaj et al., 2002; Ovecka et al., 2014), while MtMPK3 (AtMPK3 ortholog) and MtMPK6 (AtMPK6 ortholog) are localized in the cytosol, nucleus and to membranes (Chen et al., 2017; Ryu et al., 2017). In peanut, AhMPK3 (AtMPK3 ortholog) is located in both the cytosol and nucleus, while AhMPK6 (AtMPK6 ortholog) is present predominantly in the nucleus (Kumar et al., 2009; Kumar and Kirti, 2010). In soybean, GmMPK4a (AtMPK4 ortholog) is present in both the cytosol and the nucleus (Liu et al., 2011).

Overall, approximately one-third of the above cited plant MAPKs are only/predominantly localized in the nucleus, and two-thirds are present in both the cytosol and nucleus. Interestingly, probable MAPK orthologs do not seem to necessarily have the same subcellular localization, e.g., AtMPK6/NtSIPK/GhMPK6a/BnaMPK6/MtMPK6 localize in the cytosol and nucleus, while OsMPK6/TMPK6/SIMK/AhMPK6 are only nuclear. However, this apparent difference could also in part be due to technical differences, such as a lack of sensitivity of the approach or the use of heterologous expression systems.

It should also be noted that the nuclear localization of plant MAPKs is not limited to the MAPK level as some MAPKKs and MAPKKKs were also observed in this subcellular compartment. For instance, AtMKK4 is present in both the cytosol and nucleus (Yoo et al., 2008; Persak and Pitzschke, 2013), and AtMEKK1 was found in the cytosol, in the nucleus, at the plasma membrane and in vesicle-like structures (Miao et al., 2007; Gao et al., 2008; Yang et al., 2010).

## Nuclear relocalization of MAPKs

The localization of most MAPKs in both the cytosol and nucleus suggests a regulation of nucleocytoplasmic shuttling. In particular, MAPK nuclear relocalization may occur upon some stimuli, strongly suggesting that the substrates of the protein kinase are only or mainly nuclear.

In the context of biotic stress, the parsley MAPK PcMPK3a translocates from the cytosol into the nucleus within 3–10 min after treatment with the fungal elicitor Pep25 (Ligterink et al., 1997). The same group later provided a more complete picture demonstrating that parsley PcMPK6, PcMPK3a, and PcMPK3b indeed rapidly translocate from the cytosol into the nucleus after Pep13 fungal elicitor treatment, but that parsley PcMPK4, which is not activated by the elicitor, remains cytosolic (Lee et al., 2004). Moreover, the authors did not observe a nuclear accumulation of PcMKK5, the upstream kinase of PcMPK6 and PcMPK3a/b, following Pep13 treatment. In rice, OsBWMK1Long isoform, corresponding to the longest of the three existing splice variants, localizes predominantly in the cytosol and shuttles into the nucleus within 1–12 h after treatment with 2 mM of the defense-related signaling molecules H_2_O_2_ and SA (Koo et al., 2007).

In the context of abiotic stress, the translocation of Arabidopsis AtMPK3 and AtMPK6 from the cytosol to the nucleus was observed after 30 min of ozone treatment (Ahlfors et al., 2004). Likewise, the peanut AhMPK3, which localizes in untreated conditions in both the cytosol and nucleus, was shown to predominantly translocate into the nucleus upon 10 mM H_2_O_2_ treatment for 60 min (Kumar et al., 2009). In soybean, GMK1 (AtMPK6 ortholog) is present in the cytosol under normal conditions and shuttles into the nucleus 60 min after 300 mM NaCl treatment (Im et al., 2012). Interestingly, Ovecka et al. reported that in their inactive state alfalfa MAPKK SIMKK and MAPK SIMK (AtMPK6 ortholog) co-localize in the cytoplasm and in the nucleus and that upon 10–30 min of 250 mM salt stress part of the nuclear pool of both SIMKK and SIMK relocates to cytoplasmic compartments (Ovecka et al., 2014). This result challenges the classical model of MAPK translocation from the cytosol to the nucleus upon activation.

In the context of development, the Arabidopsis BASL protein exhibits a polarized cellular localization pattern that is necessary for the differential cell fates resulting from asymmetric cell division in stomatal development. MPK6 phosphorylates BASL which is important for its polarization and function. In a positive feedback loop, BASL polarization then promotes the shuttling into the nucleus of more activated MPK6 molecules (Zhang et al., 2016). Plant MAPK translocation to the nucleus was also observed in pollen maturation (differentiation process) and in pollen embryogenesis (proliferation process) (Coronado et al., 2002).

Conversely, several studies also report MAPKs that do not exhibit a detectable relocalization upon stimuli yet known to activate them. For instance, the Arabidopsis MPK12 is present in both the cytosol and nucleus of guard cells, and is activated within minutes by 50 μM ABA or H_2_O_2_ (Jammes et al., 2009). However, its subcellular localization is unaffected by ABA or H_2_O_2_ treatment, which suggests that MPK12 has substrates in both the cytosol and nucleus of guard cells.

We mentioned previously that the nuclear localization of plant MAPKs is not limited to the MAPK level as MAPKKs and MAPKKKs may also be present in the nucleus. This is true as well regarding nuclear relocalization upon stimulation. As an example, the Arabidopsis MKK9, which is upstream of MPK3 and MPK6 in ethylene signaling, shuttles into the nucleus in response to the ethylene biochemical precursor 1-aminocyclopropane-1-carboxylic acid (ACC) (Yoo et al., 2008).

**Figure 1:**
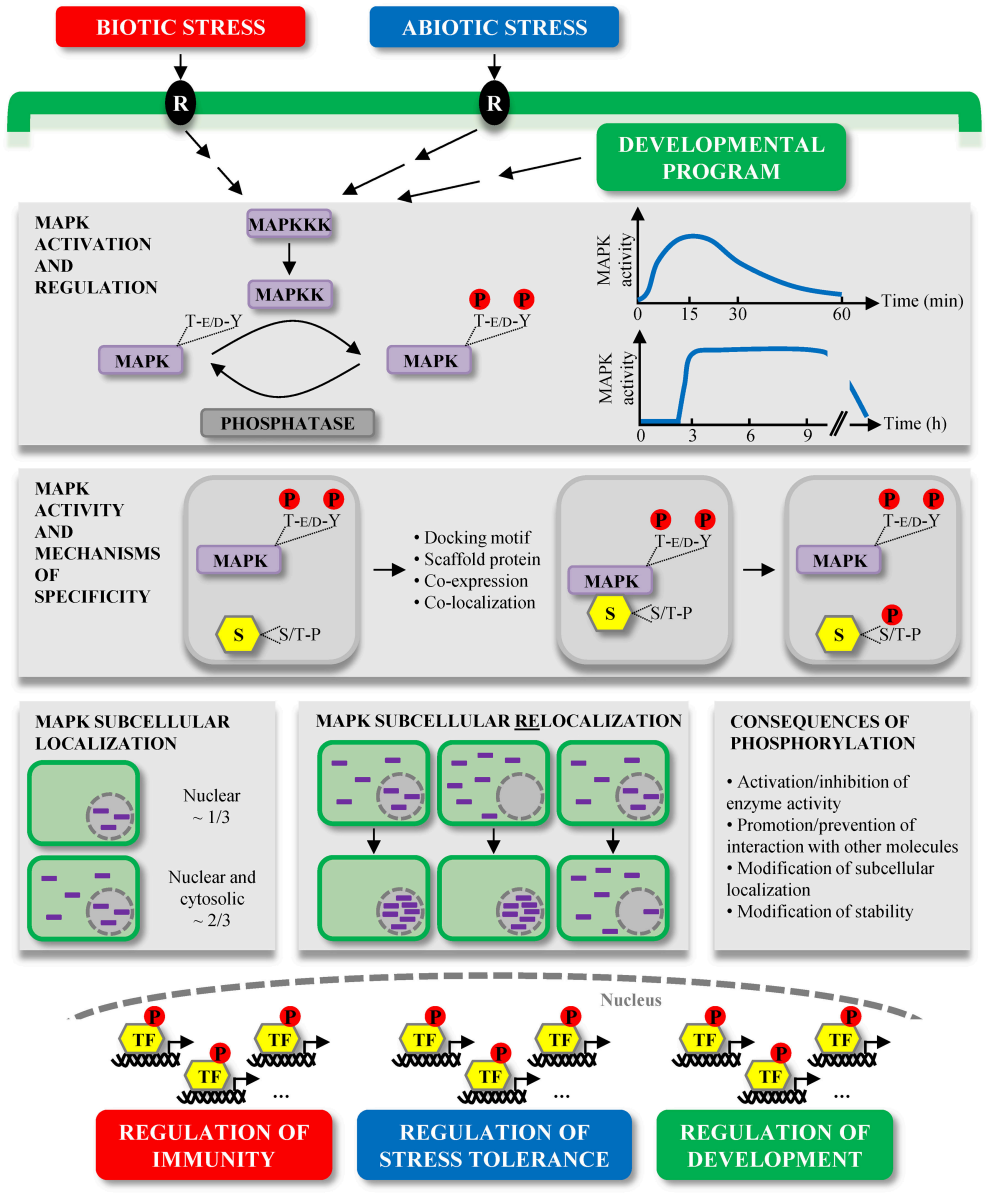
Overview of plant MAPK characteristics and signaling. MAPKs constitute the last tier of MAPK modules which are involved in the signaling of multiple environmental stresses, initially perceived by receptors (R), and developmental programs. MAPKs are activated by the dual phosphorylation of their T-E/D-Y motif in their activation loop by MAPKKs. This activation is reversible and MAPK deactivation occurs *via* their dephosphorylation by protein phosphatases. The kinetics of MAPK activation is variable depending on the stimulus, for instance in a window of several minutes during MTI, or of several hours during ETI. Once activated, MAPKs phosphorylate their substrates (S) on one or several S/T-P motifs, and besides the mandatory presence of these phosphorylation motifs, several mechanisms contribute to the substrate specificity, such as the presence of docking motifs. Substrate phosphorylation by MAPKs may have different consequences, for example their subcellular relocalization. While a few extensively characterized Arabidopsis MAPKs exhibit multiple subcellular localizations, about one third of studied plant MAPKs seem to localize only/predominantly to the nucleus, and two thirds are present in both the cytosol and nucleus. Interestingly, a few cases of MAPK subcellular relocalization were observed upon different stimuli. In almost all instances, the relocalization corresponded to a MAPK nuclear shuttling. The large majority of *bona fide* MAPK substrates identified so far are transcription factors (TF) (Table 1), phosphorylated either in the cytosol or nucleus. These phosphorylated TF then contribute to the transcriptional reprogramming, allowing the regulation of important processes in which MAPKs are involved.

Figure 1 gives an overview of the different parts developed above. In the following sections, we discuss known nuclear substrates of plant MAPKs involved in biotic stress, abiotic stress and development. For more clarity, we organized sub-sections according to plant species. As the literature is too vast to discuss all reported plant MAPK substrates as part of this review, we selected a few tens (Table 1) that were extensively studied and that benefited from both biochemical and genetic evidence, qualifying them as *bona fide* MAPK substrates. We sincerely apologize to our colleagues whose work was thus not cited.

**Table 1:** Selected nuclear substrates of plant MAPKs involved in biotic stress, abiotic stress and development that are discussed in this review.

**Stress/Species**	**Substrate**	**Substrate function**	**Substrate physiological role**	**MAPK**	**References**
**BIOTIC STRESS**					
*Arabidopsis thaliana*	MKS1	Transcription regulator	Interacts with WRKY transcription factors. Required for full salicylic acid-dependent resistance	MPK4	Andreasson et al., 2005
*Arabidopsis thaliana*	ASR3	Transcription factor	Negative regulator of MAMP-triggered immunity	MPK4	Li et al., 2015
*Arabidopsis thaliana*	PAT1	mRNA decapping machinery	*pat1* mutant exhibits effector-triggered immunity *via* the resistance protein SUMM2	MPK4	Roux et al., 2015
*Arabidopsis thaliana*	ERF104	Transcription factor	Positive regulator of basal immunity (MAMP and bacterial pathogen)	MPK6	Bethke et al., 2009
*Arabidopsis thaliana*	ERF6	Transcription factor	Positive regulator of plant immunity (necrotrophic fungal pathogen *B. cinerea*)	MPK3/MPK6	Meng et al., 2013
*Arabidopsis thaliana*	VIP1	Transcription factor	Positive regulator of plant immunity	MPK3	Djamei et al., 2007
*Arabidopsis thaliana*	TZF9	RNA binding protein	Positive regulator of MAMP-triggered immunity and resistance to *P. syringae*	MPK3/MPK6	Maldonado-Bonilla et al., 2013
*Arabidopsis thaliana*	MVQ1	Transcription regulator	Negative regulator of MAMP-triggered immunity	MPK3/MPK6	Pecher et al., 2014
*Arabidopsis thaliana*	WRKY46	Transcription factor	Positive regulator of basal plant defense	MPK3/MPK6	Sheikh et al., 2016
*Arabidopsis thaliana*	LIP5	Regulator of multivesicular body (MVB) biogenesis	Positive regulator of plant immunity (*P. syringae*)	MPK3/MPK6	Wang et al., 2014
*Arabidopsis thaliana*	WRKY33	Transcription factor	Positive regulator of plant immunity (camalexin biosynthesis)	MPK3/MPK6	Mao et al., 2011
*Oryza sativa*	OsEREBP1	Transcription factor	Positive regulator of plant immunity	BWMK1	Cheong et al., 2003
*Oryza sativa*	OsWRKY33	Transcription factor	Positive regulator of plant immunity	BWMK1	Koo et al., 2009
*Oryza sativa*	OsbHLH65	Transcription factor	Positive regulator of plant immunity	OsMPK3	Shin et al., 2014
*Oryza sativa*	OsDRB1s	Double-stranded RNA binding proteins	Participate to miRNA biogenesis	OsMPK3	Raghuram et al., 2015
*Oryza sativa*	OsWRKY30	Transcription factor	Positive regulator of plant immunity (*X. orizae*)	OsMPK7	Jalmi and Sinha, 2016
*Nicotiana tabacum*	WRKY1	Transcription factor	Enhances hypersensitive response-like cell death	SIPK	Menke et al., 2005
*Nicotiana tabacum*	SGT1	Resistance protein regulation	Required to maintain resistance proteins in an inactive but signaling-competent state	SIPK	Hoser et al., 2013
*Nicotiana tabacum*	NtWIF	Transcription factor	Positive regulator of hypersensitive response	WIPK	Yap et al., 2005
**ABIOTIC STRESS**					
*Arabidopsis thaliana*	ZAT6	Transcription factor	Positive regulator of salt and osmotic stress tolerance during seed germination	MPK6	Liu et al., 2013
*Arabidopsis thaliana*	MYB44	Transcription factor	Positive regulator of osmotic stress tolerance	MPK3	Persak and Pitzschke, 2013
*Arabidopsis thaliana*	HSFA4A	Transcription factor	Positive regulator of salt and oxidative stress tolerance	MPK3/MPK6	Perez-Salamo et al., 2014
*Arabidopsis thaliana*	AZI1	Lipid transfer protein (LTP)-related hybrid proline-rich protein (HyPRP)	Positive regulator of salt stress tolerance	MPK3	Pitzschke et al., 2014
*Arabidopsis thaliana*	LIP5	Regulator of multivesicular body (MVB) biogenesis	Positive regulator of heat and salt stress tolerance	MPK3/MPK6	Wang et al., 2015
*Arabidopsis thaliana*	HsfA2	Transcription factor	Positive regulator of heat stress tolerance	MPK6	Evrard et al., 2013
*Arabidopsis thaliana*	ICE1	Transcription factor	Positive regulator of cold stress tolerance	MPK3/MPK6	Li et al., 2017; Zhao et al., 2017
*Arabidopsis thaliana*	ERF6	Transcription factor	Regulates ROS-responsive gene transcription	MPK6	Wang et al., 2013
*Oryza sativa*	OsWRKY30	Transcription factor	Positive regulator of drought tolerance	OsMPK3/OsMPK7/OsMPK14	Shen et al., 2012
*Oryza sativa*	SUB1A1	Transcription factor	Positive regulator of submergence stress tolerance	OsMPK3	Singh and Sinha, 2016
**DEVELOPMENT**					
*Arabidopsis thaliana*	AtMAP65-1	Microtubule-associated protein	Participates to mitosis	MPK4/MPK6	Smertenko et al., 2006
*Arabidopsis thaliana*	PATL2	Binding to phosphoinositides	Participates to cytokinesis	MPK4	Suzuki et al., 2016
*Arabidopsis thaliana*	MYB44	Transcription factor	Functions in seed germination	MPK3/MPK6	Nguyen et al., 2012
*Arabidopsis thaliana*	SPCH	Transcription factor	Mediates the stomatal lineage	MPK3/MPK6	Lampard et al., 2008
*Arabidopsis thaliana*	BASL	Scaffold protein	Regulates stomatal asymmetric division	MPK3/MPK6	Zhang et al., 2015
*Arabidopsis thaliana*	MYC2	Transcription factor	Negative regulator of blue light-mediated photomorphogenesis	MPK6	Sethi et al., 2014
*Arabidopsis thaliana*	PIF3	Transcription factor	Negative regulator of red light-mediated photomorphogenesis	MPK6	Xin et al., 2018
*Nicotiana tabacum*	NtMAP65-1a	Microtubule-associated protein	Participates to phragmoplast expansion	NRK1/NTF6	Sasabe et al., 2006

## Biotic stress

### Arabidopsis MAPKs

MPK4 has a dual role in defense and regulates defense responses downstream of multiple receptors. In the absence of a pathogen, it represses innate immunity genes, but upon pathogen attack, it is needed for the full induction of a large set of defense genes. Using yeast two-hybrid screening, Andreasson et al. (2005) identified MKS1 as an MPK4 substrate. MKS1 was shown to be required for full salicylic acid (SA)-dependent resistance. MKS1 interacts with the WRKY transcription factors WRKY25 and WRKY33, which both play an important role in plant defense.

MPK4 also phosphorylates the trihelix transcriptional repressor ASR3 (Arabidopsis SH4-related 3) which negatively regulates MTI responses in Arabidopsis (Li et al., 2015). Phosphorylation of ASR3 by MPK4 enhances its DNA binding activity to suppress gene expression and plants overexpressing a phospho-mimetic form of ASR3 are compromised in MTI responses, providing further evidence how MPK4 modifies plant immune gene expression.

Arabidopsis MPK4 regulates the expression of a number of defense genes and in part this may be regulated at the level of mRNA stability. MPK4 associates with and phosphorylates PAT1, a component of the mRNA decapping machinery (Roux et al., 2015). *Pat1* mutants are dwarfed and show enhanced immunity that is dependent on the immune receptor SUMM2. Upon MAMP treatment, PAT1 accumulates in cytoplasmic processing (P) bodies which are sites for mRNA decay, linking MPK4 regulated defense gene expression to mRNA stability.

MAMPs, such as the flagellin peptide flg22, rapidly induce ethylene production. Ethylene signaling also results in the activation of MPK6 (Ouaked et al., 2003) and this process seems to be mediated by MKK9 resulting in the MAPK-dependent phosphorylation and activation of the transcription factor EIN3 (ethylene-insensitive 3) (Yoo et al., 2008). Ethylene inactivates the negative regulatory MAPKKK CTR1 (constitutive triple response 1) to activate MKK9 and MAPK activation is governed by the nuclear translocation of MKK9. In this context, Bethke et al. (2009) found the ethylene responsive transcription factor ERF104 as an interactor of MPK6. ERF104 is an important regulator of basal immunity, as *erf104* mutants showed enhanced susceptibility to a bacterial pathogen. ERF104 dissociates from MPK6 in a MAMP-dependent manner and MPK6 was found to regulate ERF104 stability in an ethylene-dependent manner.

ERF6 (ethylene response factor 6) is yet another transcription factor of Arabidopsis defense gene expression and is involved in resistance to the necrotrophic fungal pathogen *Botrytis cinerea*. MPK3/6 can phosphorylate ERF6 and thereby enhance its protein stability. Moreover, phosphomimicking ERF6 constitutively activates defense genes and confers enhanced resistance to *B. cinerea*. However, in contrast to ERF104, the regulation and function of ERF6 is independent of ethylene (Meng et al., 2013).

Apart from the role of ethylene in the regulation of defense gene expression, there is also evidence that MAPKs directly regulate ethylene production via the transcription of *ACS7, ACS8*, and *ACS11*. Here, the WRKY transcription factor WRKY33 seems to be targeted by MPK3/6 to mediate this transcriptional regulation (Mao et al., 2011; Li et al., 2012).

VIP1 (VIRE2-interacting protein1) is a bZIP transcription factor that associates during Agrobacterium transformation with the bacterial virulence protein VirE2 and helps the T-DNA to be transported into the nucleus. VIP1 is phosphorylated by MPK3 thereby inducing its nuclear relocalization and defense gene expression (Djamei et al., 2007). Phosphorylation of VIP1 also enhances its binding to the VRE (VIP1 response element) and regulates the expression of MPK3 target genes including the transcription factors *MYB44* and *MYB77* (Pitzschke et al., 2009).

The tandem zinc finger protein 9 (TZF9) is phosphorylated by MPK3/6 and needed for MTI responses (Maldonado-Bonilla et al., 2013). A *tzf9* mutant was compromised in specific MTI reactions, including reactive oxygen species (ROS) accumulation, the activation of MAPKs and the expression of several MTI marker genes. *tzf9* mutant plants show enhanced susceptibility to virulent *P. syringae* pv. *tomato* DC3000. TZF9 was localized in cytoplasmic foci that co-localize with markers of P-bodies. TZF9 can bind ribohomopolymers poly(rU) and poly(rG), suggesting a role of post-transcriptional events in plant innate immunity, such as mRNA processing or storage pathways.

The VQ motif (FxxxVQxhTG) of proteins mediates interaction with WRKY transcription factors. Yeast two-hybrid interaction screening and *in vitro* kinase assays coupled to mass spectrometry analysis identified a subset of VQ-motif-containing Arabidopsis proteins (VQPs) that are phosphorylated by MPK3/6. These proteins were called MPK3/6-targeted VQPs (MVQs) (Pecher et al., 2014). Site-directed mutagenesis of the MVQs and promoter-reporter fusion studies in protoplasts showed that flg22 induced their degradation. The MVQs interact with specific WRKY transcription factors. For MVQ1 a negative role was demonstrated in WRKY-dependent defense gene expression, including that mutation of the VQ-motif abrogates WRKY binding and causes deregulation of defense gene expression.

In a screen for WRKY transcription factors as targets of Arabidopsis MAPKs, WRKY46 was identified as a substrate of MPK3 (Sheikh et al., 2016). In protoplasts, MAMP treatment resulted in destabilization of WRKY46 and the mutation of the phosphorylation sites reduced the MAMP-triggered degradation. *WRKY46* overexpression in protoplasts enhanced basal plant defense as shown by the increased expression of the MAMP-responsive gene *NHL10*. Therefore, MAPK phosphorylation of WRKY46 provides a mechanism to regulate plant defense responses.

Multivesicular bodies (MVBs) play essential roles in many cellular processes, maintaining protein traffic by reversible membrane association of the endosomal sorting complexes required for transports (ESCRTs). Membrane dissociation of ESCRTs is catalyzed by the AAA ATPase SKD1, which is stimulated by LIP5 (LYST interacting protein 5). Arabidopsis LIP5 was found to interact with and becomes phosphorylated by MPK3/6 (Wang et al., 2014). Disruption of LIP5 has little effects on MAMP-, SA-induced defense responses but compromises basal resistance to *P. syringae* which is dependent on its interaction with SKD1. Mutation of MPK phosphorylation sites in LIP5 does not affect interaction with SKD1 but reduces the LIP5 stability and compromises its function. Moreover, pathogen infection increases formation of MVBs in a LIP5-dependent manner, indicating that MPK3/6 regulates the localization of defense molecules by MVBs.

Antimicrobial compounds also play critical roles in plant immunity. In crucifers, phytoalexins and glucosinolate derivatives are important components in defense against pathogens. MPK3/6 are important for the induction of camalexin, the major phytoalexin in Arabidopsis. Phosphorylation of WRKY33 by MPK3/6 was found to be essential for the induction of camalexin synthesis and camalexin biosynthesis genes in Arabidopsis. In *wrky33* mutants, the induction and production of camalexin was compromised (Mao et al., 2011). MPK3/6 was also found to affect the accumulation of extracellular thiocyanates which are derived from the IGS indole glucosinolate pathway (Xu et al., 2016). MPK3/6 promoted I3G (indole-3-yl-methylglucosinolate) synthesis and its conversion to 4mi3g (4-methoxyindole-3-yl-methylglucosinolate). By targeting MYB51/122, MPK3/6 control IGS biosynthesis, and via ERF6-mediated expression of *CYP81F2* and *IGMT1/2*, the two MAPKs regulate the conversion of I3G to 4MI3G.

### Rice MAPKs

Plant MAPKs are classified into two subtypes, either having a TEY or TDY motif in their activation loop. In rice, 15 proteins have been classified as MAPKs (Hamel et al., 2006). A rice D-type MAPK with a TDY phosphorylation motif BWMK1 was shown to phosphorylate the transcription factor OsEREBP1, which binds to and enhances the DNA-binding activity of OsEREBP1 to the GCC box (AGCCGCC) which is found in a number of pathogenesis-related (*PR*) gene promoters. Overexpression of *BMWK1* in tobacco plants results in enhanced levels of SA and H_2_O_2_ and *PR* genes, including hypersensitive response (HR)-like cell death (Cheong et al., 2003). OsBWMK1 is localized in the nucleus and mediates *PR* gene expression by activating the OsEREBP1 transcription factor. OsBWMK1 phosphorylates and thereby activates WRKY transcription factors, as shown for OsWRKY33 (Koo et al., 2009). Phosphorylation of WRKY33 enhances its DNA-binding activity to the W-box element (TTGACCA) of several *PR* gene promoters. Moreover, transient co-expression of *OsBWMK1* and *OsWRKY33* in Arabidopsis protoplasts enhances SA-dependent expression of a reporter gene that is mediated by a W-box element.

Shin et al. (2014) studied the TEY-type rice MAPK named OsMPK3 and reported phosphorylation of the nuclear basic helix–loop–helix transcription factor OsbHLH65 which specifically binds to E-box promoter cis-elements. OsMPK3 and OsbHLH65 were both induced by treatments with rice blast (*Magnaporthe grisea*), brown planthopper (*Nilaparvata lugens*), and the defense-related hormones methyl-JA or SA. These results suggest that OsMPK3 can contribute to defense signaling by phosphorylating the transcription factor OsbHLH65.

MicroRNA (miRNA) biogenesis is a complex process which notably requires double-stranded RNA binding (DRB) proteins. The rice genome encodes for eight DRBs. Raghuram et al. studied the *OsDRB* transcript expression suggesting their involvement in different stress responses (Raghuram et al., 2015). Interestingly, OsMPK3 interacts with and phosphorylates several OsDRB1 isoforms. The authors also obtained similar results in Arabidopsis. Overall the results suggest that MPK3 plays an important role in the regulation of miRNA level (Raghuram et al., 2015).

Group C MAPKs have been poorly characterized. Recently, the group C MAPK OsMPK7 and its upstream MAPKK OsMKK3 were shown to positively regulate resistance against *Xanthomonas oryzae* infection, known to cause leaf blight disease in rice (Jalmi and Sinha, 2016). In addition, the transcription factor OsWRKY30 interacts with and is phosphorylated by OsMPK7. OsWRKY30 is also involved in the resistance to *X. oryzae*, suggesting that a module composed of OsMKK3-OsMPK7-OsWRKY30 contributes to leaf blight disease resistance in rice (Jalmi and Sinha, 2016).

### Tobacco MAPKs

In tobacco, the MPK6 ortholog SIPK (salicylic acid-induced protein kinase) is activated by various biotic and abiotic treatments and overexpression of *SIPK* triggers cell death. In a targeted yeast two-hybrid approach, the tobacco transcription factor WRKY1 was identified as a potential substrate of SIPK (Menke et al., 2005). WRKY1 is phosphorylated by SIPK, resulting in enhanced DNA-binding activity to a W box element derived from the tobacco chitinase gene *CHN50*. Co-expression of *SIPK* and *WRKY1* in *Nicotiana benthamiana* enhances cell death induction, suggesting that SIPK mediates HR-like cell death via the regulation of WKRY1.

Resistance (R) proteins directly or indirectly sense pathogen effectors inside of host cells and are usually composed of Nucleotide-Binding and Leucine-Rich Repeat domains. SGT1 (Suppressor of G2 allele of SKP1) is required to maintain R proteins in an inactive but signaling-competent state. *Nicotiana tabacum* N protein belongs to the Toll-Interleukin Receptor (TIR)-NB-LRR class of R proteins and confers resistance to Tobacco Mosaic Virus (TMV). Using mass spectrometry, confocal microscopy and pathogen assays Hoser et al. (2013) showed that SIPK phosphorylates SGT1 and that its phosphorylation state determines its nuclear accumulation and N-mediated resistance to TMV. These data suggest that nucleocytoplasmic partitioning of N protein is determined by MAPK-dependent SGT1 phosphorylation.

Wounding of plants can be caused by mechanical stress or by herbivore and insect attack and is therefore a stress that bridges abiotic and biotic responses. Hence, WIPK (wound-induced protein kinase) also functions during pathogen responses in tobacco and phosphorylates WIPK interacting factor (NtWIF) (Yap et al., 2005; Chung and Sano, 2007). Overexpression of *NtWIF* in tobacco plants enhanced while silencing suppressed the HR. NtWIF contains a B3 DNA binding domain, which recognizes the auxin-responsive element (ARE) TGTCTC. NtWIF recognizes ARE motifs and regulates 49 stress-responsive genes, and NtWIF phosphorylation by WIPK enhances its transactivation activity.

## Abiotic stress

Abiotic stresses pose an important problem in agriculture. The dissection of the signaling pathways mediating abiotic stress tolerance is an essential step to develop more resistant plant species. In various plant species, a number of MAPKs induce responses to numerous abiotic stresses and the targets of these MAPKs are slowly emerging.

### Arabidopsis MAPKs

#### Salt, osmotic stress and ABA

C2H2-type zinc finger proteins (ZFPs) are involved in various plant responses to abiotic stress. ZAT6, an Arabidopsis C2H2-type ZFP, regulates root development and nutrient stress responses. However, it also plays a role in the regulation of abiotic stress responses. Salt and osmotic stress induces *ZAT6* expression and *ZAT6* overexpressing plants show improved seed germination under these stress conditions. ZAT6 interacts and is phosphorylated at two sites by MPK6 *in vitro* and *in planta*. Overexpression of a phosphorylation site mutant compromised the enhanced salt and osmotic stress tolerance observed with the wild type ZAT6. These results suggest MPK6 phosphorylation of ZAT6 is required to execute its role during seed germination under salt and osmotic stress conditions (Liu et al., 2013).

The transcription factor MYB44 was identified in a yeast two-hybrid screen for interacting partners of MKK4. MAMP-induced expression of *MYB44* is regulated by the MPK3-targeted bZIP transcription factor VIP1 (Pitzschke et al., 2009). MPK3 interacts with and phosphorylates MYB44 at Ser145. The mutation of Ser145 in MYB44 to the non-phosphorylatable Ala145 or the phosphomimetic D145 did not change its subcellular localisation, its homo-dimerization nor its DNA-binding activity (Persak and Pitzschke, 2013). However, overexpression of *MYB44* resulted in enhanced tolerance to osmotic stress and plants that were slightly more sensitive to ABA. In contrast, and similar to *mpk3* plants, overexpression of *MYB44* plants with an Ala145 mutation resulted in hypersensitivity to abiotic stress.

In plants, heat shock factors (HSFs) are not only implicated in heat stress but also in a number of abiotic stresses, as shown by estradiol-induced tolerance to salt and oxidative stress of HSFA4A. In contrast, inactivation of HSFA4A results in hypersensitivity to salt stress in Arabidopsis. *HSFA4A* expression decreases, while an *hsfa4* knock out enhances levels of H_2_O_2_ and lipid peroxides. Overexpression of *HSFA4A* alters expression of a large set of oxidative stress genes. Protein interaction assays show that HSFA4A homodimerization is reduced by replacement of three conserved cysteine residues by alanine residues. MPK3/6 interact with and phosphorylate HSFA4A on three sites, whereby Ser309 seems to be the major residue (Perez-Salamo et al., 2014). Activation of MPK3/6 led to the transcriptional activation of the *HSP17.6A* (heat shock 17.6A) gene and an Ala309 variant of HSFA4A strongly reduces the transcriptional induction of *HSP17.6A*. These data suggest that abiotic stress responses by HSFA4A are mediated by MPK3/6 signaling in Arabidopsis.

Another MPK3 substrate that is involved in salt stress is AZI1. AZI1 is a lipid transfer protein (LTP)-related hybrid proline-rich protein (HyPRP) that is phosphorylated by MPK3 *in vitro*. Co-immunoprecipitation and bimolecular fluorescence complementation assays show that AZI1 interacts with MPK3. *AZI1* overexpressing plants are more tolerant whereas *azi1* knock out mutants are hypersensitive to salt stress. Furthermore, *AZI1* overexpression in the *mpk3* genetic background partially suppresses the hypersensitive phenotype of *mpk3* plants under salt stress conditions (Pitzschke et al., 2014).

LIP5 is an AAA ATPase in MVB biogenesis and a positive regulator of SKD1 (Suppressor of K+ Transport Growth Defect1). LIP5 is also a substrate of MPK3/6 which plays an important role in the plant immune system (see section above). Wang et al. (2015) showed that the LIP5-regulated MVB pathway is also important in plant responses to abiotic stress. *lip5* mutants are compromised in both heat and salt stress tolerance. The function of LIP5 in plant abiotic stress depends on its interaction with SKD1. *lip5* mutants accumulate ubiquitinated protein aggregates under heat stress and increased Na^+^ and Cl^−^ levels under salt stress. LIP5 mediates abiotic stress-induced increases of endocytic vesicles and MVBs. Moreover, LIP5 is involved in the salt-induced increases of intracellular ROS levels. Basal levels of LIP5 phosphorylation by MAPKs and LIP5 stability increase upon salt stress, and mutation of the MAPK-targeted phosphorylation sites in LIP5 reduces its stability and compromises its function. These results suggest that MPK3/6 regulates the MVB pathway via LIP5 phosphorylation and plays a critical role in biotic and abiotic stress responses.

#### Heat and cold stress

HSFs are key regulators of plant responses to several abiotic stresses, but for heat stress, the transcription factor HsfA2 plays a dominant role. *In vitro* and *in vivo* evidence shows that MPK6 specifically targets HsfA2 during heat stress (Evrard et al., 2013). Activation of MPK6 results in enhanced complex formation with HsfA2 and HsFA2 phosphorylation induces intracellular relocalization. Interestingly, protein kinase and phosphatase inhibitors revealed that HsfA2 protein stability is also regulated by phosphorylation, but independently of MPK6.

Previous studies showed that the MEKK1-MKK2-MPK4/6 pathway positively regulates responses to cold and freezing in Arabidopsis (Teige et al., 2004). MPK4 and MPK6 are activated by cold stress in plants by MKK2 (Teige et al., 2004). Mutants in *mkk2* exhibit increased sensitivity to freezing, whereas constitutive activation of MKK2 enhances freezing tolerance by increasing expression of *CBF* genes (Teige et al., 2004). CRLK1 interacts with MEKK1, leading to MAPK activation and freezing tolerance (Yang et al., 2010). Recent publications show that, in contrast to the MEKK1-MKK2-MPK4 cascade, which positively regulates cold tolerance, the MKK5-MPK3/MPK6 cascade negatively regulates cold responses by promoting the degradation of ICE1 through phosphorylation. MPK3/6 phosphorylation of ICE1 inhibits its transcriptional activity and facilitates ubiquitination-mediated ICE1 degradation, thereby negatively regulating plant freezing tolerance and *CBF* expression (Li et al., 2017; Zhao et al., 2017).

#### ROS

ROS have been shown to be both important signaling molecules that mediate many developmental and physiological responses, but until now, the transcriptional mechanisms regulating ROS-responsive genes are still little understood. Recently, ROS-responsive cis-acting elements (ROSEs) were identified from the promoters of genes up-regulated by ROS in Arabidopsis. ERF6 (APETALA2/ethylene-responsive element binding factor 6) could bind specifically to the ROSE7/GCC box. Co-expression of *ERF6* enhanced luciferase activity driven by ROSE7. The deficient mutants of *ERF6* showed growth retardation and higher sensitivity to photo-damage. MPK6 interacts with and phosphorylates ERF6 at both S266 and S269. ERF6 phosphorylation affects ERF6 protein levels resulting in changes in ROS-responsive gene transcription. These data link the regulation of ROS-responsive gene transcription under oxidative stress or fluctuating light conditions to MAPK signaling (Wang et al., 2013).

### Rice MAPKs

#### Drought

Both the WRKY transcription factors and MAPKs have been shown to regulate gene expression in response to biotic and abiotic stresses in Arabidopsis. In rice, Shen et al. (2012) showed that OsWRKY30 interacted with OsMPK3, OsMPK4, OsMPK7, OsMPK14, OsMPK20-4, and OsMPK20-5, and could be phosphorylated by OsMPK3, OsMPK7, and OsMPK14. Overexpression of *OsWRKY30* in rice dramatically increased drought tolerance, but not when the phosphorylation sites were replaced by alanine. This can be explained by the fact that the transcriptional activation of OsWRKY30 was also impaired in the alanine substituted mutant.

#### Submergence stress

Recently, a link between MAPK signaling and the tolerance to submergence stress was uncovered. SUB1A is an ethylene response factor-like protein that regulates a number of responses during submergence of rice. Singh and Sinha (2016) showed that MPK3 is activated by submergence and that MPK3 phosphorylates SUB1A. Furthermore, SUB1A1 binds to the *MPK3* promoter and regulates its expression in a positive regulatory loop upon submergence stress. These data suggest a key role for SUB1A and MPK3 in acclimation of rice seedlings to submergence stress.

## Development

### Cell division

Cell division depends on microtubule dynamics and organization. The tobacco MAPK module, which includes the MAPK NRK1/NTF6, positively regulates cytokinesis at the level of the phragmoplast, preceeding the synthesis of the cell plates at cell division. NRK1/NTF6 phosphorylates the T579 in the microtubule-associated protein NtMAP65-1a which reduces its microtubule bundling activity (Sasabe et al., 2006). During M phase, NRK1/NTF6 is activated and levels of phosphorylated NtMAP65-1 increase concomitant with their accumulation at the phragmoplast. Overexpression of phosphosite mutants of NtMAP65-1a delays progression of M phase and phragmoplast expansion.

Cytokinesis in Arabidopsis is similarly regulated by the formation of the cell plate and a MAPK pathway that is highly homologous to that identified in tobacco and includes the ANP MAPKKKs, the MAPKK MKK6 and the MAPK MPK4. Mutants in these genes are dwarfed and have characteristic cytokinesis defects, such as immature cell plates. MKK6 activates MPK4 in protoplasts and MPK4 kinase activity is high in dividing cells but not in *mkk6* mutants. MPK4 protein is localized to the expanding cell plates in dividing cells (Kosetsu et al., 2010). AtMAP65-1 was shown to be phosphorylated during mitosis by CDK and MAPK (Smertenko et al., 2006) and expression of non-phosphorylatable AtMAP65-1 negatively affects mitosis.

Recently, using two-dimensional difference gel electrophoresis to identify phosphorylated proteins from *mpk4* mutant plants, Suzuki et al. (2016) could identify the SEC14 domain protein PATELLIN2 (PATL2) as a substrate of MPK4. As MPK4, PATL2 is concentrated at the cell division plane. PATL2 has binding affinity for phosphoinositides which are altered after phosphorylation by MPK4, suggesting a role for the MAPK cascade in the formation of cell plates via a regeneration of membranes during cytokinesis.

### Germination

The phytohormones ABA and gibberellic acid (GA) play antagonistic functions in seed germination and seedling development. In Arabidopsis, the transcriptional regulator MYB44 functions in seed germination (Nguyen et al., 2012). In dry seeds, *MYB44* transcript levels are high but decrease during germination. This decrease during germination is inhibited by the GA biosynthesis inhibitor paclobutrazol (PAC) and overexpression of *MYB44* results in increased sensitivity of seed germination to ABA or PAC. MYB44 is phosphorylated by MPK3/6 and *myb44* mutant phenotype by PAC can be suppressed by wild type *MYB44* but not by a MYB44 version that lacks the MAPK-targeted phosphorylation sites. These results indicate that MAPK-dependent phosphorylation of MYB44 is required for its functional role during germination.

### Stomatal development

Stomata modulate gas exchange between plants and the atmosphere and play critical roles in plant growth and stress adaptation. The stomatal lineage and its subsequent asymmetric divisions in Arabidopsis are mediated by the basic helix-loop-helix transcription factor SPEECHLESS (SPCH). The entry into the stomatal lineage is controlled by phosphorylation of SPCH by either the GSK3-like kinase BIN2 (BRASSINOSTEROID INSENSITIVE 2) and MPK3/6, promoting the degradation of the lineage determinant SPCH (Lampard et al., 2008).

Cell polarization is linked to cell fate determination during asymmetric division of plant stem cells. In Arabidopsis, BASL is polarized to control stomatal asymmetric division. A positive-feedback loop between BASL and the MPK3/6 pathway constitutes a module at the cell cortex. Phosphorylated BASL functions as a scaffold and recruits the MAPKKK YODA and MPK3/6 to the cell cortex (Zhang et al., 2015) and the activation of MPK3/6 reinforces the feedback loop by phosphorylating BASL and inhibiting stomatal fate by phosphorylating SPCH. Therefore, the polarization of the signaling feedback module BASL-MAPK connects cell polarity to cell fate differentiation during asymmetric plant stem cell division.

### Photomorphogenesis

Upon light exposure, plants shift to photomorphogenic growth, during which chlorophyll is synthesized, cotyledons develop and hypocotyl growth slows down. In Arabidopsis, the bHLH transcription factor MYC2 negatively regulates blue light-mediated photomorphogenic growth (Yadav et al., 2005). Blue light activates the MKK3-MPK6 module and this activation is regulated by MYC2 (Sethi et al., 2014). Besides, MPK6 interacts with and phosphorylates MYC2. These results suggest that a feedback regulatory mechanism involving MKK3-MPK6 and MYC2 occurs in response to blue light.

The bHLH transcription factors phytochrome-interacting factors (PIFs) negatively regulate photomorphogenesis. Upon exposure to light, PIFs are phosphorylated and degraded. Recently, Xin et al. reported the involvement of Arabidopsis MKK10-MPK6 module in regulating cotyledon opening upon red light exposure (Xin et al., 2018). Moreover, MPK6 interacts with and phosphorylates PIF3 leading to its degradation. Collectively, the results of Xin et al. suggest that a module composed of MKK10-MPK6-PIF3 regulates seedling photomorphogenesis in response to red light (Xin et al., 2018).

## Conclusions and future prospects

The majority of currently known (nuclear) MAPK substrates are targets of Arabidopsis MPK3, MPK4, and MPK6, or their probable orthologs in other plant species. This observation is true whatever the context, biotic stress, abiotic stress or development. Identifying the functions of the other MAPKs, notably via the identification of their substrates, thus constitutes an important challenge to better understand signaling networks. Likewise, most of known (nuclear) MAPK substrates were identified in Arabidopsis. It is expected nonetheless that more and more crop MAPK substrates will be identified in the future, thanks notably to the huge progress made these last years in genome sequencing. Translational research, such as targeted approaches on probable crop orthologs of Arabidopsis MAPK substrates, will likely allow developing more resistant/tolerant plant species.

Most currently known *bona fide* plant MAPK substrates are nuclear and more precisely are transcription factors. This underlines the major role of plant MAPKs as regulators of transcriptional reprogramming, notably allowing within minutes the implementation of adapted responses to environmental constraints. For instance, we showed the strong involvement of Arabidopsis MPK3, MPK4, and MPK6 in the transcriptional reprogramming upon MAMP treatment (Frei Dit Frey et al., 2014).

Currently, known functions of plant MAPKs are supposed to only rely on their protein kinase activities. However, interestingly, the mechanism of action of mammalian ERK2 (and very probably ERK1) goes beyond its role as a protein kinase. ERK2 was indeed shown to have kinase-independent functions, for instance by promoting activation of proteins by simple direct interaction, by acting as a transcriptional regulator through DNA binding and by disrupting protein complexes (Rodríguez and Crespo, 2011). It would be interesting to investigate if plant MAPKs also have kinase-independent functions.

Besides, plant MAPKs are believed to work as monomers. In contrast, it is known that phosphorylation and scaffold proteins stimulate ERK homodimerization, and that ERK homodimers have cytoplasmic substrates while nuclear substrates are phosphorylated by ERK monomers (Casar et al., 2008). Interestingly, there is also some evidence that different rice MAPKs can directly interact with each other, suggesting that not only MAPK homo- but also MAPK hetero-dimers might play a role in signaling (Sheikh et al., 2013). Therefore, it would be interesting to investigate if such mechanisms also apply to other plant MAPKs and what their functional role is in the respective signaling pathway.

While the dogma is that dual phosphorylation of the T-loop by MAPKK is required for MAPK activation (Anderson et al., 1990; Payne et al., 1991), some other rare mechanisms were also reported. For instance, in the yeast MAPK Hog1/p38 the threonine residue of the TxY motif is essential for the biological activity of the MAPK while the tyrosine residue is not (Bell and Engelberg, 2003). Likewise, the mammalian p38α MAPK can be activated by direct interaction with the protein TAB1 or through Y323 phosphorylation by T cell receptor-proximal tyrosine kinases and both ways induce the cis-autophosphorylation of its T-loop (residues Thr180 and Tyr182) (Ge et al., 2002; Salvador et al., 2005). Interestingly, similar mechanisms have been reported for plant MAPKs. Indeed, Arabidopsis MPK8 can be activated through direct interaction with calmodulins in a Ca^2+^-dependent manner and moreover the activation does not require MPK8 autophosphorylation (Takahashi et al., 2011). Also in rice, the calcium-dependent protein kinase CPK18 is able to activate MPK5 (AtMPK3 ortholog) by phosphorylation, however this phosphorylation mainly occurs on two conserved threonine residues in the N-terminal region (Thr14 and Thr32) of MPK5, but not on its TxY motif (Xie et al., 2014). The observation of these unconventional MAPK activation mechanisms strongly suggests that others may exist as well.

The current model of MAPK signaling is represented by the MAPK module where a MAPKKK activates one/several MAPKK(s) which in turn activate(s) one/several MAPK(s) that finally phosphorylate(s) its/their substrates. However, data suggest that MAPKKKs could also have other substrates than MAPKKs, thus forming signaling shortcuts. Indeed, Arabidopsis MEKK1 can directly interact with the transcription factor WRKY53 and bind the *WRKY53* promoter (Miao et al., 2007). The authors also reported the *in vitro* phosphorylation of WRKY53 by MEKK1, but no *in planta* evidence has supported this result yet. Likewise, Arabidopsis MAP3K16 interacts with and phosphorylates *in vitro* the negative regulator of ABA response ABR1, but here as well *in planta* evidence is lacking (Choi et al., 2017). Arabidopsis MAP3K20 directly interacts with MPK18 and phosphorylates MPK18 *in vitro* on the TDY motif of its activation loop (Benhamman et al., 2017). This signaling shortcut is suggested to occur in parallel to a classical MAPK module, constituted of MAP3K20, MKK3, and an unknown MAPK, in cortical microtubule function.

Besides individual PTM events, such as phosphorylation, PTM crosstalk constitutes an additional level in the regulation of protein properties (Hunter, 2007). Protein phosphorylation is likely the most abundant PTM found in eukaryotes but other PTMs are also frequent, such as acetylation, methylation, ubiquitination and SUMOylation (Minguez et al., 2012). While numerous studies were made to uncover plant phosphoproteomes, other PTMs have been poorly explored. Substantial efforts will be required to identify the whole set of protein PTMs, thereby allowing the study of PTM crosstalk and the uncovering of new regulatory mechanisms.

## Author contributions

All authors listed have made a substantial, direct and intellectual contribution to the work, and approved it for publication.

### Conflict of interest statement

The authors declare that the research was conducted in the absence of any commercial or financial relationships that could be construed as a potential conflict of interest.
